# The relationship between emotional inhibition, emotional deprivation, failure, vulnerability to harm schema, and severity of symptoms among patients with obsessive-compulsive disorder

**DOI:** 10.1186/s12888-024-06119-x

**Published:** 2024-10-15

**Authors:** Mira Naguib Abdelrazek, Ayman Mohamed El-Ashry, Heba Mohamed Abdelaal

**Affiliations:** 1 Lecturer of psychiatric & Mental Health Nursing, faculty of nursing, Alexandria University, Alexandria, Egypt; 2 Assistant professor of psychiatric & Mental Health Nursing, faculty of nursing, Alexandria University, Alexandria, Egypt

**Keywords:** Emotional self-awareness, Failure, Vulnerability to harm, Obsessive-compulsive disorder

## Abstract

**Objective:**

Assess the relationship between emotional inhibition, emotional deprivation, failure, vulnerability to harm schema, and severity of symptoms among obsessive-compulsive disorder.

**Methods:**

A descriptive correlational study was conducted on 30 patients with obsessive-compulsive disorder who were recruited to complete the Yale-Brown Obsessive-Compulsive Scale, Young Schema Questionnaire-Long Form.

**Results:**

The results of the study indicate that 66.6% of the 30 subjects exhibited high levels of OCD symptom severity, with a mean score of 33.20 ± 4.67. In terms of early maladaptive schemas, 60% of subjects displayed severe emotional deprivation, 66.7% showed severe emotional inhibition, and 83.3% had severe failure schemas. A strong positive correlation was found between failure schemas and OCD symptoms (*r* = 0.697, *p* < 0.001). However, what truly impressed the researchers was the predictive power of the failure schema. It was the only significant predictor of OCD symptoms (Beta = 0.992, *p* < 0.001), explaining 55.2% of the variance.

**Conclusion:**

The study underscores the crucial influence of early maladaptive schemas on the severity of obsessive-compulsive disorder symptoms in individuals with obsessive-compulsive disorder. It proposes that considering early maladaptive schemas, such as emotional self-awareness, failure, and vulnerability to harm, can aid in gauging the severity of obsessive-compulsive disorder symptoms. Moreover, the study’s findings are of significant importance to mental health professionals, researchers, and individuals involved in the treatment and management of obsessive-compulsive disorder, as they provide a deeper understanding of the condition and suggest practical approaches for its management.

## Introduction

According to the World Health Organization (WHO), Obsessive-Compulsive Disorder (OCD) was the fourth most common mental illness worldwide in 2019, following drug abuse, social anxiety, and depression. OCD is characterized by obsessions, which are intrusive, unwelcome thoughts, desires, or images, and compulsions, repetitive actions, or mental processes carried out in response to these obsessions or according to specific rules. Common compulsions include washing, checking, counting, and verbally repeating phrases [1]. The Diagnostic and Statistical Manual of Mental Disorders (DSM-V, 2013) reports that the global 12-month prevalence of OCD is between 1.1% and 1.8% [2]. In Egypt, Ghanem et al. (2009) found that 4.75% of respondents experienced anxiety disorders, including OCD [3]. Untreated OCD poses a significant risk for severe mental, familial, and social dysfunction and can increase the likelihood of developing additional psychiatric conditions [4].

Early maladaptive schemas (EMSs), as defined by Young (1990, 2003, and 2014), are enduring cognitive patterns formed by childhood trauma and unmet emotional needs. These schemas substantially influence the development of symptoms, which include various mental health disturbances such as anxiety, depression, and OCD [5–7]. While the authors refer to “symptoms,” they mean general psychological symptoms, which can manifest as emotional and behavioral issues, but in the context of OCD, these schemas can directly relate to OCD-like symptoms. Schema Therapy (ST) aims to foster change by promoting adaptive coping mechanisms and offering corrective experiences to heal negative childhood memories, enhancing psychological well-being [5, 8, 9]. Schema therapy has been proven effective in addressing a wide range of psychological issues, though further research is required to clarify its specific impact on OCD symptoms.

Young et al. (2003) proposed a model of eighteen EMSs categorized into five domains. The first domain, Disconnection and Rejection, includes schemas such as emotional deprivation, abandonment, mistrust/abuse, defectiveness/shame, and social isolation/alienation. The second domain, Impaired Autonomy and Performance, consists of schemas involving feelings of inadequacy, dependence, enmeshment, and vulnerability to harm. The third domain, Impaired Limits, addresses grandiosity, entitlement, and a lack of self-discipline. The fourth domain, Other-Directedness, covers subjugation, approval-seeking, and self-sacrifice. The fifth domain, Over-Vigilance and Inhibition, relates to negativity, emotional inhibition, perfectionism, and punitiveness [6, 7]. This framework offers a comprehensive understanding of how EMSs may contribute to a variety of psychological issues, including OCD.

In the context of OCD, patients predominantly exhibit schemas such as emotional deprivation, vulnerability to harm or illness, failure, and emotional inhibition. These specific schemas are hypothesized to be more prevalent in individuals with OCD, as they align with the characteristic emotional and cognitive patterns observed in the disorder. Research supports the presence of these schemas in OCD, though further empirical studies are needed to fully establish their prevalence [6, 7]. According to Young’s Schema Theory [6], emotional inhibition deficits, linked to emotional deprivation and inhibition schemas, can exacerbate OCD symptoms. Due to cognitive distortions, OCD patients often experience an exaggerated fear of disasters and an inability to prevent them. Halford et al. (2002) note that these fears are marked by excessive worry, compulsive rituals, and constant reassurance-seeking [10].

OCD patients frequently exhibit alexithymia or difficulty identifying and expressing their emotions [11]. In addition to these schemas, OCD patients often display vulnerability to harm and failure schemas, reflecting a perceived inability to prevent disasters and a pervasive sense of impending personal failure. The failure schema involves believing in inevitable personal inadequacy, while the vulnerability schema reflects an inflated fear of harm [12].

Schema therapy has shown promise in reducing these maladaptive schemas and improving emotional inhibition in OCD patients, although it remains unclear whether these improvements directly alleviate OCD symptoms. Some evidence suggests that lowering emotional deprivation and inhibition schemas can help patients gain better emotional control, but more research is needed to confirm the therapy’s overall effectiveness in treating OCD [6, 7]. The emotional deprivation schema refers to a perceived lack of emotional support and nurturing, while the emotional inhibition schema represents the belief that emotions must be suppressed to avoid negative consequences. These schemas contribute to the difficulty that many OCD sufferers experience in recognizing and processing their emotional experiences [11].

Furthermore, OCD patients commonly display cognitive distortion where individuals believe their thoughts alone can influence outcomes, regardless of any real-world cause-and-effect relationship. This belief leads to compulsive behaviors like checking, washing, avoidance, and symmetry-related rituals to prevent perceived harm. According to Young’s Schema Theory, these thought patterns are linked to the failure and vulnerability schemas, which reinforce cognitive biases in OCD patients [6, 7].

There is a significant link between EMSs, mainly the failure schema, and the severity of OCD symptoms. This finding highlights the potential of schema therapy to improve emotional awareness and reduce OCD-related symptoms, making it a valuable tool in clinical practice. Nurses and mental health professionals can use schema therapy to help patients address specific schemas and alter maladaptive thought patterns. OCD characteristics such as excessive caution, perfectionism, difficulty making decisions, and an exaggerated fear of catastrophe are strongly associated with vulnerability and failure schemas. Schema therapy can help patients change their catastrophic thinking and avoidance behaviors to better cope with their perceived threats [12].

### The aim of this study is to

- Assess the level of emotional inhibition, emotional deprivation, failure, vulnerability to harm, or illness schemas among patients with OCD.
- Assess the relationship between emotional inhibition, emotional deprivation, failure, vulnerability to harm schema, and severity of symptoms among OCD.

### Research questions

- What are the levels of emotional inhibition, emotional deprivation, failure, vulnerability to harm, or illness schemas among patients with OCD?
- What is the relation between emotional inhibition, emotional deprivation, failure, vulnerability to harm schema, and severity of symptoms among OCD?

## Measure and procedure

### Research design

This study followed a descriptive correlational design, meticulously adhering to STROBE guidelines, ensuring the thoroughness and reliability of our research.

### Setting

The study was carried out at the University Hospital’s psychiatric outpatient clinics, which are connected to Alexandria University’s Faculty of Medicine. The clinics provide free therapeutic services for individuals with neuropsychiatric illnesses. These services include mental health assessment, diagnosis, prescription administration, and, for some patients, psychotherapy. The clinics are open from 8 a.m. to 2 p.m. on Sundays, Mondays, Tuesdays, and Thursdays.

### Participants

Based on the outpatient records of the setting mentioned above, the number of patients diagnosed with OCD who visit the psychiatric outpatient clinics per week ranges between 1 and 2 patients, 4–8 patients per month, and 12–24 patients / 3 months (the psychiatric outpatient clinic’s records of the Main University Hospital 2022–2023). The research subjects comprised the total number of patients who visited the psychiatric outpatient clinics for 3 months—about 30 patients diagnosed with OCD according to the DSM-5 with no comorbidity.

### Tools of the study

#### Yale-Brown obsessive-compulsive scale (Y-BOCS)

It was created by Goodman et al. (1989) and adapted by Storch et al. (2010). It was a widely recognized instrument for evaluating the severity of OCD symptoms and treatment outcomes. The scale features a semi-structured interview with 10 core items, split into five for assessing obsessive thoughts and five for compulsions. Each item is scored from 0 (no symptoms) to 4 (severe symptoms), resulting in a total global severity score ranging from 0 to 40. Total scores classify individuals into subclinical OCD as corresponding to a total score of 0 to 13, mild OCD to 14 to 21, moderate OCD to 22 to 29, and severe OCD to 30 to 40, offering a detailed assessment of symptom severity [13, 14]. Reports indicate that the Y-BOCS has a test-retest correlation value of 0.84, an inter-rater correlation coefficient of 0.98, and a Cronbach’s alpha of 0.89 [13, 15].

#### Young Schema questionnaire–long form, Third Edition (YSQ-L3)

The Young et al. (2003) questionnaire assesses 18 early maladaptive schemas using 232 items organized into five domains: disconnection and rejection, poor autonomy/performance, impaired limits, other-directedness, and over-vigilance and inhibition. Subscales that measure various elements of maladaptive schemas related to emotional and psychological functioning are included in each domain [5, 16]. In the current study, four EMS subscales associated with OCD were evaluated based on the work of Young, Klosko, and Weishaar (2003; 2014) [5, 6]. These subscales include emotional deprivation (items 1–9), failure (items 69–77), vulnerability to harm or illness (items 93–104), and emotional inhibition (items 143–151). Participants were rated 39 statements using a 6-point Likert scale, ranging from 1 (“Completely untrue of me”) to 6 (“Describes me perfectly”). Scores are then calculated, with higher scores indicating greater levels of Early Maladaptive Schemes. The Cronbach’s alpha for the YSQ-L3 and the test-retest correlation coefficients for its domains were 0.83–0.96 and 0.50–0.80, respectively [17].

A socio-demographic and clinical data-structured interview schedule for patients with OCD was used to elicit data about the patients studied, such as age, sex, marital status, educational level, occupation, living situation, and area of residence. It also covered clinical data such as illness duration, starting treatment date, medications presently taken, and medication compliance.

### Ethical considerations

Approval was sought from the Ethical Research Committee at the Faculty of Nursing, Alexandria University in Egypt. Written permission to conduct the research was obtained from relevant authorities. After explaining the research’s aim, informed written consent was obtained from recruited patients or their accompanying persons. Measures were in place to ensure and respect the confidentiality of collected data. Patient privacy was considered and respected throughout the research process. Patients voluntarily participated, and their right to withdraw from the research was respected.

### Validity and reliability

The Arabic versions of the Young Schema Questionnaire-Long Form and the Yale-Brown Obsessive-Compulsive Scale were examined by a panel of five experts in psychiatric nursing and psychology. With a Lawshe Content Validity Ratio of more than 0.99, they concluded that the content of the instruments was legitimate. The specialists guaranteed the accuracy, precision, and relevance of five patients who matched the inclusion criteria, which were used to evaluate internal consistency using Cronbach’s alpha test technique. The results showed high reliability with values of 0.77 for Tool III (YSQ-L3) and 0.72 for Tool II (Y-BOCS).

### Pilot study

After obtaining official permissions, a pilot study involving 10 patients diagnosed with OCD was conducted to ensure the clarity and applicability of tools (Tool II—Y-BOCS and Tool III—YSQ-L3). The pilot study confirmed that the tools were clear, well-understood, and applicable, requiring no modifications. Subjects from the pilot study were excluded from the actual research.

### Data collection

The researcher visited the El-Amiry University Hospital thrice weekly to select available patients for three months. Patients’ medical charts were reviewed to confirm their diagnosis and ensure they fulfilled the inclusion criteria. 30 patients with OCD (both males and females) were recruited based on the screening of patients’ charts. One to two patients were interviewed daily to explain the study’s purpose, establish rapport, and assess the severity of OCD symptoms and EMSs using Tools I, II, and III. The interviews with Tools I, II, and III took about 45–60 min, with individual differences leading to variations in session durations. After each patient interview, manual calculation of the scores for Tools II and III was performed to identify the severity of OCD symptoms and the level of EMSs.

### Statistical analysis of the data

Data analysis for the study was done with IBM SPSS software, version 23.0. Customarily distributed quantitative variables were correlated using the Pearson coefficient. Regression analysis was used to forecast how EMSs affect the intensity of OCD symptoms. At a standard alpha level of 0.05, the results’ significance was evaluated at 5%.

## Results

Patients’ socio-demographic and clinical characteristics show that 43.3% were male, and the subjects’ ages ranged between 23 and 39 years, with the majority (63.3%) falling in the 31–40 age group. Regarding marital status, 73.3% of the subjects were single. In terms of educational background, 80% had a university-level education. Notably, 76.6% of the subjects were unemployed, and all subjects (100%) resided in urban areas. Regarding birth order, 43.3% were middle children, and 56.6% came from families with 3 to 6 members. Regarding income, 70% of the subjects reported sufficient income. The majority (76.6%) had no previous hospital admissions. Additionally, 73.3% of the subjects had a positive family history of mental illness (see Table 1).

**Table 1:** Distribution of the studied subjects according to their socio- demographic and clinical characteristics

Patients’ socio-demographic Characteristics	Total subjects: 30	
	No	%
Sex		
Male	**13**	**43.33**
Female	**17**	**56.66**
**Age (years)**		
20–30	**11**	**36.66**
31–40	**19**	**63.33**
**Marital status**		
Single	**22**	**73.33**
Married	**8**	**26.66**
**Level of education**		
Secondary	**6**	**20**
University	**24**	**80**
**Work status**		
Un-employed	**23**	**76.66**
Employed	**7**	**23.33**
**Place of Residence**		
Urban	**30**	**100**
**Birth order**		
First chil	**11**	**36.66**
Middle child	**13**	**43.33**
Last child	**6**	**20**
**Family size**		
1–3	**13**	**43.33**
4–6	**17**	**56.66**
**Income**		
Not enough	**9**	**30**
Enough	**21**	**70**
**Cohabitation (with family)**		
With husband /wife	**12**	**40**
With father and mother	**18**	**60**
**Duration of illness**		
Less than 5 years	**21**	**70**
More than 5 years	**9**	**30**
Mean ± SD.	**3.4615 ± 2.9612**	
**Previous admission**		
Yes	**7**	**23.44**
No	**23**	**76.66**
**Family history**		
Positive	**8**	**26.66**
Negative	**22**	**73.33**

All OCD patients (100%) received a combination of pharmacotherapy and psychotherapy. Regarding medication use, 23.3% of subjects received only antipsychotics, and 53.3% received both antidepressants and antipsychotics. Meanwhile, 13.3% were on antidepressants only. Notably, all subjects (100%) reported compliance with their medication regimen (see Table 2).

**Table 2:** Distribution of the studied subjects according to their treatment modalities

Patients’ treatment modalities	Total subjects: 30	
	No	%
**Type of treatment**		
Medication only	**0**	**0**
Medication and psychotherapy sessions	**30**	**100**
ECT	**3**	**10**
**Compliance**		
Compliance	**30**	**100**
Non-compliance	**0**	**0**
**Medication used**		
Aripiprazole (Antipsychotic)	**7**	**23.33**
Clozapex. Aripiprazole (Antipsychotic)	**3**	**10**
Faverin or Philozac, aripiprazole (antidepressants, Antipsychotic)	**16**	**53.33**
Philozac (antidepressants)	**4**	**13.33**

About 66.6% of patients were classified as having a high level of OCD symptom severity, while the remaining 33.3% fell into the severe category. The mean OCD severity score was 33.20 ± 4.67, with scores ranging from 24 to 40 (see Table 3).

**Table 3:** Distribution of the studied subjects according to obsessive compulsive symptoms severity level

OCD severity level	Studied subjects (No = 30)	
	No	%
**Severe level** **Moderate level**	10 20	33.3 66.6
**Min –max** **Mean ± SD**	24–40 33.20 ± 4.67	

Subclinical 0–13 mild OCD 14–21 moderate 22–29 severe OCD 30–40

The majority of patients (60%) showed severe levels of emotional deprivation, while 66.7% had severe levels of emotional inhibition. Failure schemas were particularly prevalent, with 83.3% of the subjects falling into the severe category. Additionally, 63.3% of subjects exhibited severe vulnerability to harm or illness, reflecting significant emotional and cognitive difficulties within the studied group (see Table 4).

**Table 4:** Descriptive statistics for early maladaptive schemas

EMSs	Descriptive statistics		Levels	Studied subjects (No = 30)	
	Mean	Std. deviation		No	%
**Emotional Deprivation**	**40.43**	**11.39**	Mild Moderate Severe	2 10 18	6.7 33.3 60
**Emotional Inhibition**	**40.86**	**8.41**	Mild Moderate Severe	1 9 20	3.3 30 66.7
**Failure**	**43.96**	**6.69**	Mild Moderate Severe	0 5 25	0 16.7 83.3
**Vulnerability to harm or illness**	**52.40**	**13.14**	Mild Moderate Severe	4 7 19	13.3 23.3 63.3

A strong positive correlation was found between failure schemas and OCD symptoms (*r* = 0.697, *p* < 0.001), indicating that higher levels of the failure schema are associated with increased OCD symptom severity. Emotional inhibition also showed a positive correlation with OCD symptoms, though to a lesser extent (*r* = 0.445, *p* = 0.014). In contrast, emotional deprivation and vulnerability to harm had weaker, non-significant correlations with OCD symptoms (see Table 5).

**Table 5:** Of correlation between early maladaptive schemas and OCD symptoms

	OCD symptoms	
EMSs	Pearson correlation	*R*
**Emotional Deprivation**	0.086	0.318
**Emotional Inhibition**	0.014^*^	0.445^*^
**Failure**	< 0.001^*^	0.697^*^
**Vulnerability to harm or illness**	0.919	-0.019

R person correlation *p significant at ≤ 0.001.

A linear regression analysis predicting OCD symptoms based on EMSs is shown in Table 6. The model explains 55.2% of the variance in OCD symptoms (R² = 0.552), which is statistically significant (F = 7.688, *p* < 0.001). Of the schemas analyzed, the failure schema was the only significant predictor of OCD symptoms (B = 0.353, Beta = 0.992, t = 4.348, *p* < 0.001), indicating that higher failure schema scores lead to increased OCD symptom severity. Emotional inhibition (B = -0.122, *p* = 0.185), emotional deprivation (B = -0.006, *p* = 0.958), and vulnerability to harm (B = 0.126, *p* = 0.124) did not significantly predict OCD symptoms.

**Table 6:** Linear Regression Analysis of early maladaptive schemas predict OCD symptoms

EMSs	B	Beta	t	*p*	95% CI	
					LL	UL
**Emotional inhibition**	-0.122	-0.298	-1.364	0.185	-0.307	0.062
**Emotional deprivation**	-0.006	-0.008	-0.053	0.958	-0.232	0.220
**Failure**	0.353	0.992	4.348^*^	< 0.001^*^	0.186	0.520
**Vulnerability to harm**	0.126	0.227	1.591	0.124	-0.037	0.290
R^2^ = 0.552, adjusted R^2^ = 0.531, F = 7.688^*^, *p* > 0.001^*^						

F, p: f and *p* values for the model; R^2^: Coefficient of determination; B: Unstandardized Coefficients.

Beta: Standardized Coefficients; t: t-test of significance; LL: Lower limit UL: Upper Limit

*: Statistically significant at *p* ≤ 0.05

## Discussion

The long-lasting mental health condition known as OCD has a significant impact on daily functioning. In the DSM-5, OCD is categorized under “obsessive-compulsive and related disorders.” It is characterized by recurrent, distressing thoughts called obsessions, which are often accompanied by anxiety. These obsessions lead to repetitive behaviors or mental acts, known as compulsions, aimed at reducing the anxiety or discomfort caused by the obsessions. As a chronic condition, OCD affects a substantial portion of the population and can be challenging to manage.

In this study, 60% of OCD patients were found to be in the “Severe level” category for the Emotional Deprivation Schema, indicating a high prevalence of maladaptive schemas, such as emotional deprivation, emotional inhibition, failure, and vulnerability to harm or illness. These findings align with research by Sij et al. (2018), which linked emotional dysregulation to early interpersonal trauma, suggesting that traumatic childhood experiences may contribute to the development of maladaptive schemas [17]. These schemas, particularly emotional deprivation and inhibition, are associated with difficulties in expressing emotions and seeking support, as individuals with these schemas struggle to recognize and express their basic needs. This perspective is consistent with Young’s schema model, which emphasizes how EMSs shape emotional and interpersonal functioning [17, 18].

The study also highlights the severity of OCD symptoms, with a notable percentage of patients falling into the severe category for both OCD and specific schemas, such as emotional deprivation. One of the studies reviewed used the Yale-Brown Obsessive Compulsive Scale (Y-BOCS) to assess OCD severity, focusing on compulsive behaviors and obsessive thoughts as indicators of severe OCD. This further supports the notion that many individuals with severe OCD may also exhibit severe levels of the Emotional Deprivation Schema [19]. Another study on children and adolescents with moderate to severe OCD similarly categorized severity using the Y-BOCS scale, reinforcing the variability of OCD severity among patients and its potential links to EMSs like emotional deprivation [20].

Regarding emotional inhibition, 66.7% of the study participants fell into the “Severe level” category for this schema, and the findings suggest a positive association between OCD symptoms and emotional inhibition. Patients with high levels of emotional inhibition tend to suppress their emotions to avoid social rejection or shame, a phenomenon described by cognitive pathology theory, which suggests that maladaptive schemas are activated when individuals face emotional challenges [5, 21, 22]. The relationship between OCD and emotional regulation deficits is further explored in studies showing that OCD patients often suppress emotions instead of using healthier reappraisal strategies, a finding that aligns with emotional inhibition [23].

Furthermore, research into emotional processing in OCD highlights that individuals with OCD frequently experience intense negative emotions, such as anxiety, fear, and disgust, in response to their obsessive thoughts, further supporting the connection between OCD and emotional inhibition [24].

One of the most significant findings of this study is the strong positive correlation between the Failure Schema and OCD symptoms. Multiple studies have confirmed the association between EMSs, particularly the Failure Schema, and the severity of OCD symptoms. For instance, research investigating schema therapy’s impact on emotional inhibition and vulnerability in OCD patients highlights the prominence of the Failure Schema, which contributes significantly to the presentation of OCD symptoms [16]. This study’s findings are consistent with other research showing that the Failure Schema has a powerful link to OCD severity [25]. Additionally, a comparison of EMSs between OCD and panic disorder revealed that maladaptive schemas, such as mistrust/abuse, social isolation, and vulnerability to harm, are also standard in OCD patients, with the Failure Schema demonstrating the most substantial correlation with OCD symptoms [26].

The clinical implications of these findings are significant, yet the exact nature of the relationship between OCD and EMSs remains unclear. It is uncertain whether these schemas contribute to the development of OCD or result from the disorder. Research, including cross-sectional studies, offers mixed evidence: Some studies suggest that maladaptive schemas and dysfunctional beliefs directly influence OCD symptom severity, while others do not support this link. This ambiguity has important treatment implications. Understanding whether schemas are a cause or an exacerbating factor is essential for determining the focus of therapeutic interventions, such as schema therapy or cognitive-behavioral therapy. Further, longitudinal studies are necessary to clarify this relationship and inform clinical practice [17, 27].

The study also found weaker correlations between the Vulnerability to Harm and Emotional Deprivation schemas and OCD symptoms, consistent with research showing that while these schemas are present in OCD patients, their impact may not be as strong as other schemas, such as the Failure Schema [28]. Another study supports this finding, showing that OCD patients score higher on the Failure Schema than on the Emotional Deprivation Schema, and the relationship between vulnerability to harm and OCD symptoms is not as significant as other schemas [29]. Together, these results suggest that while the Vulnerability to Harm Schema and Emotional Deprivation Schema are present in OCD patients, their influence on OCD symptoms may be less pronounced.

### Limitations of the study

The researchers in the present study chose a limited number of patients according to the availability of the diagnosed clients with OCD; the study needs to be applied to a larger sample if possible.

### Conclusion and recommendations

The search results suggest that the Failure Schema may have a strong positive correlation with OCD symptoms, while the Vulnerability to Harm Schema may have a weaker correlation. Schema therapy can be used to modify emotional inhibition and vulnerability, which may be associated with OCD symptoms. These findings suggest that EMSs may play a role in the development and maintenance of OCD symptoms and that schema therapy may be a functional treatment approach for individuals with OCD.

### Relevance for clinical nursing practice

Findings from the search yield helpful knowledge on the connection between EMSs and OCD symptoms for nurses practicing nursing. With this knowledge, nurses can work with mental health specialists to offer OCD patients schema therapy, identify EMSs in OCD patients, and create tailored treatment programs that focus on particular schemas linked to OCD symptoms.

## Data Availability

The datasets used and/or analysed during the current study are available from the corresponding author on reasonable request.

## Abbreviations

ED: Emotional Deprivation
EI: Emotional Inhibition
EMS: Early maladaptive schemas
FA: Failure to Achieve
OCD: Obsessive Compulsive Disorder
ST: Schema Therapy
VH: Vulnerability to Harm or Illness
WHO: World Health Organization
YBOC: Yale brown obsessive-compulsive scale
YSQ: Young schema questionnaire

## References

[1] WHO. The most common psychiatric disorders. Geneva: World Health Organization. 2019. https://www.who.int/mental_health/management/en/

[2] American Psychiatric Association. Diagnostic and statistical Manual of Mental disorders DSM-V-TR. 5th ed. Arlington, VA: American Psychiatric Association; 2013.

[3] Ghanem M, Gadallah M, Meky F, Mourad S, El-Kholy G. National Survey of Prevalence of Mental Disorders in Egypt: preliminary survey. East Mediterr Health J. 2009;15(1):65–75. https://iris.who.int/handle/10665/117609 19469428

[4] Fineberg NA, Dell’Osso B, Albert U, Maina G, Geller D, Carmi L, Sireau N, Walitza S, Grassi G, Pallanti S, Hollander E. Early intervention for obsessive compulsive disorder: an expert consensus statement. Eur Neuropsychopharmacol. 2019;29(4):549–65. 10.1016/j.euroneuro.2019.02.002 30773387 10.1016/j.euroneuro.2019.02.002

[5] Young J, Klosko J. Reinventing your life: how to break free from negative life patterns. New York: Dutton; 1993.

[6] Young J, Klesko J, Weishaar M. Schema Therapy: a practitioner’s guide. New York: Guilford Press; 2003.

[7] Young J. Early maladaptive schemas – revised. New York, NY: Cognitive Therapy Center of New York; 2014.

[8] Hashemi R, Darvishzadeh K. Effectiveness of Group Schema Therapy in reducing the symptoms of Major Depression in a sample of women. Asian Soc Sci. 2016;12(6):232. 10.5539/ass.v12n6p232

[9] Basile B, Tenore K, Luppino O, Mancin F. Schema therapy mode model applied to OCD. Clin Neuropsychiatry. 2017;14(6):407–414. https://www.apc.it/wp-content/uploads/2017/12/2017-schema-therapy-mode-Basile-et-al-clinical-neuropsychiatry.pdf

[10] Halford W, Doolan S, Eadie K. Schemata as moderators of clinical effectiveness of a comprehensive cognitive behavioral program for patients with depression or anxiety disorders. Behav Modif. 2002;26(5):571–93. 10.1177/014544502236651 12375375 10.1177/014544502236651

[11] Kang JI, Namkoong K, Yoo SW, Jhung K, Kim SJ. Abnormalities of emotional awareness and perception in patients with obsessive-compulsive disorder. J Affect Disord. 2012;141(2–3):286–93. 10.1016/j.jad.2012.04.001 22542863 10.1016/j.jad.2012.04.001

[12] Bottoms L, Prat Pons M, Fineberg NA, Pellegrini L, Fox O, Wellsted D, Drummond LM, Reid J, Baldwin DS, Hou R, Chamberlain S. Effects of exercise on obsessive-compulsive disorder symptoms: a systematic review and meta-analysis. Int J Psychiatry Clin Pract. 2023;27(3):232–42. 10.1080/13651501.2022.2151474 36541901 10.1080/13651501.2022.2151474

[13] Goodman W, Price L, Rasmussen S, et al. The Yale-Brown obsessive-compulsive scale. II: validity. Arch Gen Psychiatry. 1989;46(11):1012–6. 10.1001/archpsyc.1989.01810110054008 2510699 10.1001/archpsyc.1989.01810110054008

[14] Storch EA, Rasmussen SA, Price LH, Larson MJ, Murphy TK, Goodman WK. Development and psychometric evaluation of the Yale–Brown Obsessive-compulsive scale—Second Edition. Psychol Assess. 2010;22(2):223–32. 10.1037/a0018492 20528050 10.1037/a0018492

[15] Bauer L. Getting control: overcoming your obsessions and compulsions. 2nd ed. Tehran: Arjomand; 2009. 10.5812/modernc.69656. Persian.

[16] Thiel N, Caffier B, Herbst N, Külz A, Nissen C, Hertenstein E, et al. The prediction of treatment outcomes by early maladaptive schemas and schema modes in obsessive-compulsive disorder. BMC Psychiatry. 2014;14:362. 10.1186/s12888-014-0362-0 25540106 10.1186/s12888-014-0362-0 PMC4324412

[17] Sij ZD, Manshaee G, Hasanabadi H, Nadi MA. The effects of schema therapy on emotional self-awareness, vulnerability, and obsessive symptoms among patients with obsessive-compulsive disorder. Mod Care J. 2018;15(2). 10.5812/modernc.69656

[18] Fassbinder E, Schweiger U, Martius D, Brand-de Wilde O, Arntz A. Emotion regulation in schema therapy and dialectical behavior therapy. Front Psychol. 2016;7:1373. 10.3389/fpsyg.2016.01373 27683567 10.3389/fpsyg.2016.01373 PMC5021701

[19] Cervin M, OCD Severity Benchmark Consortium, Mataix-Cols D. Empirical severity benchmarks for obsessive-compulsive disorder across the lifespan. World Psychiatry. 2022;21(2):315–6. 10.1002/wps.20984 35524607 10.1002/wps.20984 PMC9077592

[20] Luo L, Feng B, Yang S, Zhang N, Qiu S. Clinical characteristics of moderate-severe obsessive-compulsive disorder in children and adolescents in China. J Int Med Res. 2020;48(5). 10.1177/0300060520922679 10.1177/0300060520922679 PMC7273799 32458715

[21] Voderholzer U, Schwartz C, Thiel N, Kuelz AK, Hartmann A, Scheidt CE, et al. A comparison of schemas, schema modes and childhood traumas in obsessive-compulsive disorder, chronic pain disorder and eating disorders. Psychopathology. 2014;47(1):24–31. 10.1159/000348484 23689753 10.1159/000348484

[22] Williams AD, Thompson J, Andrews G. The impact of psychological distress tolerance in the treatment of depression. Behav Res Ther. 2013;51(8):469–75. 10.1016/j.brat.2013.05.005 23787227 10.1016/j.brat.2013.05.005

[23] Ferreira S, Couto B, Sousa M, Vieira R, Sousa N, Pico-Perez M, et al. Stress influences the effect of obsessive-compulsive symptoms on emotion regulation. Front Psychiatry. 2021;11:594541. 10.3389/fpsyt.2020.594541 33551866 10.3389/fpsyt.2020.594541 PMC7854917

[24] Thorsen AL, Hagland P, Radua J, Mataix-Cols D, Kvale G, Hansen B, et al. Emotional Processing in Obsessive-compulsive disorder: a systematic review and Meta-analysis of 25 functional neuroimaging studies. Biol Psychiatry Cogn Neurosci Neuroimaging. 2018;3(6):563–71. 10.1016/j.bpsc.2018.01.009 29550459 10.1016/j.bpsc.2018.01.009 PMC5994188

[25] Akbarikia H, Gasparyan K. Schema and locus of control as predictors of obsessive-compulsive disorder. Iran J Psychiatry. 2012;7(4):170–175. https://pubmed.ncbi.nlm.nih.gov/23408715 PMC3570575 23408715

[26] Kwak KH, Lee S. A comparative study of early maladaptive schemas in obsessive–compulsive disorder and panic disorder. Psychiatry Res. 2015;230. 10.1016/j.psychres.2015.11.015 10.1016/j.psychres.2015.11.015 26599390

[27] TEMPLE SD. Schema Therapy: A Practitioner’s Guide. Am J Psychiatry. 2003;160(11). 10.1176/appi.ajp.160.11.2074-a. 2074-a-2075.

[28] Kizilagac F, Cerit C. Assessment of early maladaptive schemas in patients with obsessive-compulsive disorder. Dusunen Adam J Psychiatry Neurol Sci. 2019;32(1):14. 10.14744/DAJPNS.2019.00003

[29] Kim JE, Lee SW, Lee SJ. Relationship between early maladaptive schemas and symptom dimensions in patients with obsessive-compulsive disorder. Psychiatry Res. 2014;215(1):134–40. 10.1016/j.psychres.2013.07.036 23962740 10.1016/j.psychres.2013.07.036

